# MRI should be routine for all patients with localized prostate cancer? | *Opinion: No*

**DOI:** 10.1590/S1677-5538.IBJU.2016.06.04

**Published:** 2016

**Authors:** Joel B. Nelson

**Affiliations:** 1 Department of Urology, University of Pittsburgh School of Medicine, Pittsburgh, PA, USA

**Keywords:** Magnetic Resonance Imaging, Prostatic Neoplasms, Diagnosis, Watchful Waiting

One of the basic principles of medical care is that a diagnostic test should inform a clinical decision. If the test is uninformative, it is not useful; if no decision is to be made then a diagnostic test is not necessary. Indeed, performing a diagnostic test when it adds nothing to the decision-making process is not only a waste of healthcare resources, it is potentially harmful, leading to incorrect conclusions or more unnecessary testing. From this perspective, how could mp-MRI potentially inform the initial management of localized prostate cancer?

Men who are candidates for active surveillance based on low-risk prostate cancer (cT1c, PSA<10, Grade Group 1 (Gleason 3+3=6)) may be harboring a higher grade tumor that eluded the initial biopsy, particularly if it is anteriorly placed. mp-MRI has the promise of detecting this potentially more serious cancer and avoiding the risk of inappropriate observation. Some have argued men with low-risk prostate cancer with a “normal” mp-MRI (PI-RADS 1) have very little risk of cancer progression. The promise of mp-MRI to provide a better risk assessment in men considering active surveillance is alluring.

Unfortunately, the evidence that mp-MRI used in the initial assessment of clinically localized prostate cancer to improve the outcome of men undergoing active surveillance is lacking. The slow growth of prostate cancer means that even higher grade cancer will become clinical evident, with or without an MRI. Metastatic or lethal prostate cancer in the largest active surveillance cohorts–mostly selected without mp-MRI–are rare events (1). It appears readily available clinical features like PSA, grade and stage are adequate in almost all cases to determine who can safely start down the path of active surveillance. Although a “normal” mp-MRI may be reassuring to a patient and/or their physician it actually adds very little to the decision-making process.

The local staging of prostate cancer based on a digital rectal exam (DRE), which only assesses the posterior aspect of the gland, could be enhanced by the whole-gland assessment provide by mp-MRI. Certainly it is better to know the location, size and extent of the cancer before considering treatment. This information can determine whether the cancer is surgically resectable and guide surgical approach, particularly in the decision to preserve neurovascular tissues. It could also guide a focal therapeutic approach. The improved staging provided by mp-MRI make it an attractive test to obtain in the initial assessment of clinically localized prostate cancer.

Unfortunately, mp-MRI is not as nearly as attractive as promised in prostate cancer staging. First, prostate cancer is distinguished by multifocality within the gland. The accuracy of mp-MRI to find all the prostate cancers is woeful. Using the gold-standard of whole-mount histopathology compared to preoperative mp-MRI, the overall sensitivity of mp-MRI to detect tumors was 47% (2). Sensitivity improved to 72% for tumors over 1 cm or with Gleason score ≥7, and up to 80% for the index tumors. Prostate cancers less than 5 mm in diameter are essentially invisible on mp-MRI. As a staging test, mp-MRI is only useful for lesions it can detect: the remainder the gland remains a clinical black box. Second, accurate staging requires defining the extent of an infiltrating cancer. The accuracy of mp-MRI to locate the edge of a cancer is awful. It systematically underestimates histologically determined tumor boundaries. This is particularly true for the most clinically concerning cancers, those with a high imaging suspicion score and high Gleason score (3). This is why focal therapy based on mp-MRI is not nearly as focal as it could be, as a 9 mm treatment margin is advocated with focal therapies to ensure successful treatment of the entire tumor (3). Others have shown prostate cancer foci are an average 11 mm longer and have a volume 3 times greater than estimated by MRI (4). Again, as a staging test, mp-MRI comes up short. Third, the defining the presence of extracapsular extension (ECE) can directly influence a therapeutic approach. Here, mp-MRI has consistently demonstrated high specificity, in the 90% range (5). If one suspects ECE on mp-MRI there is a high likelihood it will be present on final pathological assessment of the specimen. Unfortunately, mp-MRI has very poor sensitivity to detect histological ECE, roughly 50% (5). There is no assurance ECE is not present when the mp-MRI doesn't detect it. Overall, as a staging test, mp-MRI is far from attractive: “homely” would be a better descriptor.

Zealots for mp-MRI in the management of prostate cancer have suggested it has the ability to grade the cancer, or at least distinguish between low-risk (Gleason score 6) and higher-risk (Gleason score 7, 8 and 9) tumors. Although low-risk tumors have higher ADCs than higher risk tumors, the ADC overlap between Gleason score 6 and Gleason score 7 cancers is significant: roughly half of Gleason scores 7 cancers have ADCs that are indistinguishable from Gleason score 6 cancers (6). Furthermore, the ranges of ADC between roughly half of Gleason score 7 and all Gleason score 8 and 9 cancers are overlapping. By far, the most accurate assessment of grade is by histology, not radiology.

Another problem of mp-MRI for prostate cancer is the significant challenge of accurate interpretation. The literature is replete with examples of the variability of radiologists to assess prostate cancer using mp-MRI (7, 8). The learning curve appears to be steep and long: this is not a skill acquired easily. Unless prostate mp-MRI becomes a standard test in all cases of prostate cancer–a dubious goal given the points articulated above–it is reasonable to question whether there will be enough informative cases to train the world of radiologists to interpret them. Furthermore, using the current mp-MRI technology, it seems the limits of this test have been reached. The visual and cognitive challenge for a radiologist to accurately define prostate cancer will only be as good as the mp-MRI images that routinely fail to detect, define and characterize prostate cancer.

Value in healthcare has been defined as the outcome divided by the cost (9). Outcomes of men with prostate cancer must be demonstrably improved by introducing the additional expense of mp-MRI for that technology to be considered of value. Frankly, I see almost no evidence mp-MRI has improved the outcome of population of patients with prostate cancer.

Accepting that mp-MRI may not be useful in every man with clinically localized prostate cancer, isn't there a role in selected patients, particularly when it may directly influence a surgical approach? In my opinion, the answer to this question must be based on an honest assessment of the outcomes of one's own surgical series and clinical experience as opposed to practice patterns the field. This is admittedly the least scientifically approach to answering the question, but it is also the most applicable to the next patient presenting to me as a surgeon. If including mp-MRI in the initial assessment of that patient will improve his eventual outcome, it should be obtained: if it will not, then it adds nothing.

**Figure 1:**
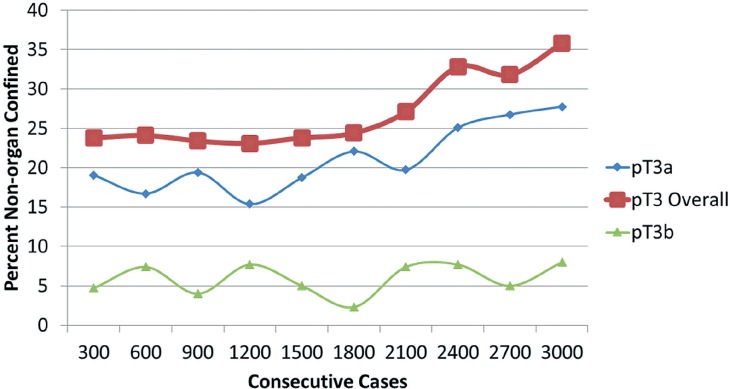
Percent of non-organ confined prostate cancer in 3000 consecutive patients undergoing open radical retropubic prostatectomy by a single surgeon (JBN).

**Figure 2:**
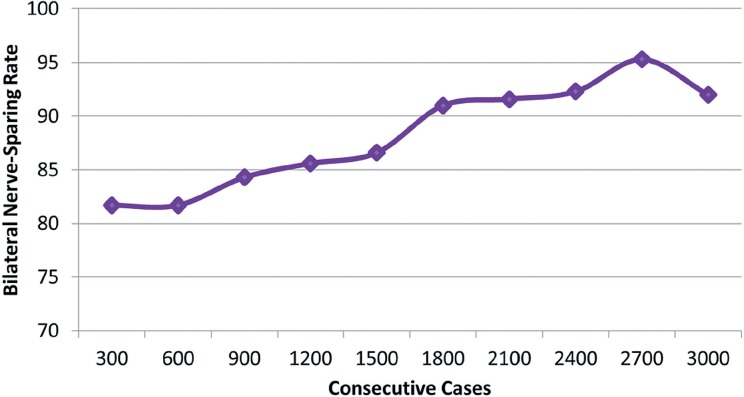
Frequency of bilateral nerve-sparing procedures in 3000 consecutive patients undergoing open radical retropubic prostatectomy by a single surgeon (JBN).

**Figure 3:**
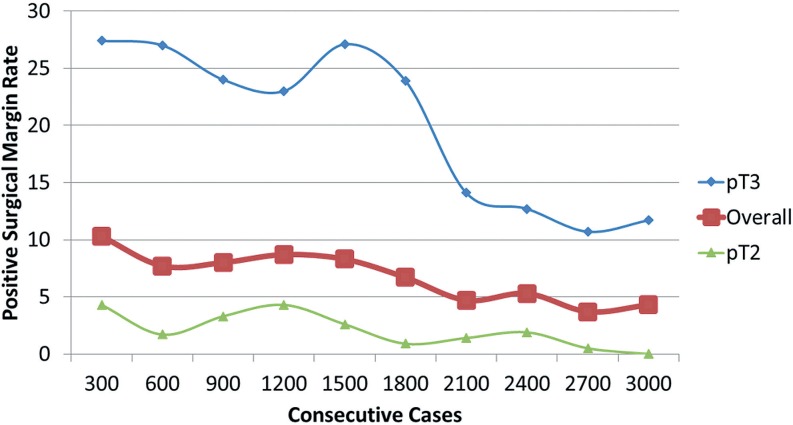
Rates of positive surgical margins in 3000 consecutive patients undergoing open radical retropubic prostatectomy by a single surgeon (JBN).

I only perform open radical retropubic prostatectomies, so my thoughts about surgical approach may not be applicable to those using a minimally-invasive approach. First, decisions about the resectability of a prostate cancer have reliably been based on DRE. Unresectable tumors–those invading the levators or distal urethra–are obvious. An mp-MRI would only confirm what is already known by physical exam. Very rarely have I encountered cancer grossly invading the bladder intraoperatively; although mp-MRI may have been useful in avoiding surgery in those handful of cases, it certainly does not justify obtaining imaging even in selected cases. Furthermore, since I routinely widely resect the bladder neck, any positive margin at that location is microscopic and are invisible to mp-MRI. Second, the need to perform a wider resection of the neurovascular bundle in an area of suspected ECE is not a mystery. Preoperative DRE and inter-operative assessments are rarely misleading. Third, even if mp-MRI suggests ECE, clinical T3 disease does not equate to surgical futility. Men with pT3 disease can still be cured with radical prostatectomy and mp-MRI should not dissuade taking that approach. Indeed, in my series of 3000 consecutive open radical prostatectomies, the percent of non-organ confined cancers has increased significantly (Figure-1). Despite more locally invasive disease, the frequency of bilateral nerve-sparing procedures remains over 90% (Figure-2). How? Because for the last 1200 cases, the overall positive surgical margin rate has been under 5% (6.7% for the entire series) (Figure-3). With experience and meticulous attention visual and haptic cues, the positive surgical margin rate for pT3 diseases has been less than 15% for the last 1200 cases. Only a fraction of these patients underwent an mp-MRI prior to surgery (obtained by another physician) and in no case did the result of that study influence the operative approach. Based on this experience, I tell my patients who are candidates for a radical prostatectomy that mp-MRI plays no role in the clinical decision-making process.

I hope my comments will quickly become of historic interest only, as better imaging techniques overcome the current short-comings of mp-MRI and allow precise cancer localization and characterization. This will make whole-gland focused therapies largely obsolete and allow us to observe the majority of prostate cancers that are of no threat.
